# People-centred integrated care in urban China

**DOI:** 10.2471/BLT.18.214908

**Published:** 2018-10-01

**Authors:** Xin Wang, Xizhuo Sun, Stephen Birch, Fangfang Gong, Pim Valentijn, Lijin Chen, Yong Zhang, Yixiang Huang, Hongwei Yang

**Affiliations:** a School of Public Health, SUN Yat-sen University, No. 74, Zhongshan 2nd Road, Yuexiu District, Guangzhou 510080, China.; b Shenzhen Luohu Hospital Group, Shenzhen, China.; c Centre for the Business and Economics of Health, University of Queensland, Brisbane, Australia.; d Department of Health Services Research, Care and Public Health Research Institute (CAPHRI), Faculty of Health, Medicine and Life Sciences, Maastricht University, Maastricht, Netherlands.; e China National Health Development Research Centre, Beijing, China.

## Abstract

In most countries, the demand for integrated care for people with chronic diseases is increasing as the population ages. This demand requires a fundamental shift of health-care systems towards more integrated service delivery models. To achieve this shift in China, the World Health Organization, the World Bank and the Chinese government proposed a tiered health-care delivery system in accordance with a people-centred integrated care model. The approach was pioneered in Luohu district of Shenzhen city from 2015 to 2017 as a template for practice. In September 2017, China’s health ministry introduced this approach to people-centred integrated care to the entire country. We describe the features of the Luohu model in relation to the core action areas and implementation strategies proposed and we summarize data from an evaluation of the first two years of the programme. We discuss the challenges faced during implementation and the lessons learnt from it for other health-care systems. We consider how to improve collaboration between institutions, how to change the population’s behaviour about using community health services as the first point of contact and how to manage resources effectively to avoid budget deficits. Finally, we outline next steps of the Luohu model and its potential application to strengthen health care in other urban health-care systems.

## Résumé

Dans la plupart des pays, la demande de soins intégrés pour les personnes atteintes de maladies chroniques augmente à mesure que la population vieillit. Cette demande nécessite une réorientation majeure des systèmes de soins de santé vers des dispositifs de prestation de services plus intégrés. Pour effectuer cette réorientation en Chine, l'Organisation mondiale de la Santé, la Banque mondiale et le gouvernement chinois ont proposé un système de soins de santé à plusieurs niveaux selon un dispositif de soins intégrés axés sur l'être humain. Cette approche a été utilisée pour la première fois dans le district de Luohu de la ville de Shenzhen de 2015 à 2017 en tant que modèle de pratique. En septembre 2017, le ministère chinois de la Santé a appliqué à l'ensemble du pays ce dispositif de soins intégrés axés sur l'être humain. Nous décrivons les caractéristiques du modèle de Luohu par rapport aux principaux domaines d'action et aux stratégies de mise en œuvre proposées et nous résumons les données extraites d'une évaluation des deux premières années du programme. Nous examinons les difficultés rencontrées lors de la mise en œuvre et les leçons tirées de ces difficultés pour d'autres systèmes de soins de santé. Nous réfléchissons aux moyens d'améliorer la collaboration entre les institutions, de changer le comportement de la population concernant l'utilisation des services de santé des collectivités comme premier point de contact et de gérer efficacement les ressources pour éviter les déficits budgétaires. Enfin, nous décrivons les prochaines étapes à suivre dans le cadre du modèle de Luohu et son application potentielle pour renforcer les soins de santé dans d'autres systèmes urbains de soins de santé.

## Resumen

En la mayoría de los países, la demanda de atención integrada para las personas con enfermedades crónicas aumenta a medida que la población envejece. Esta demanda requiere un cambio fundamental de los sistemas de atención sanitaria hacia modelos de prestación de servicios más integrados. Para lograr este cambio en China, la Organización Mundial de la Salud, el Banco Mundial y el gobierno chino propusieron un sistema escalonado de prestación de servicios sanitarios de acuerdo con un modelo de atención integrada centrada en las personas. El enfoque se introdujo en el distrito de Luohu de la ciudad de Shenzhen de 2015 a 2017 como modelo para la práctica. En septiembre de 2017, el Ministerio de Salud de China introdujo este enfoque de atención integrada centrada en las personas en todo el país. Se describen las características del modelo de Luohu en relación con las áreas centrales de acción y las estrategias de implementación propuestas y se resumen los datos de una evaluación de los dos primeros años del programa. Se exponen los desafíos enfrentados durante la implementación y las lecciones aprendidas de la misma para otros sistemas de atención sanitaria. Se considera cómo mejorar la colaboración entre las instituciones, cómo cambiar el comportamiento de la población sobre el uso de los servicios sanitarios comunitarios como primer punto de contacto y cómo gestionar eficazmente los recursos para evitar déficits de presupuesto. Por último, se esbozaron los próximos pasos del modelo de Luohu y su posible aplicación para fortalecer la atención sanitaria en otros sistemas urbanos de atención sanitaria.

## ملخص

في معظم الدول، يتزايد الطلب على خدمات الرعاية المتكاملة للأشخاص المصابين بأمراض مزمنة كلما ازداد عدد السكان. ويتطلب ذلك تحولا أساسيا في نظم الرعاية الصحية باتجاه نماذج لتقديم الخدمات أكثر تكاملا. ولتحقيق هذا التحول في الصين، اقترحت كل من منظمة الصحة العالمية، والبنك الدولي، والحكومة الصينية نظاماً لتقديم الرعاية الصحية المتدرجة وفقاً لنموذج للرعاية المتكاملة المرتكزة على الأشخاص. ظهر هذا النهج في منطقة لوهو بمدينة شنتشن من عام 2015 إلى عام 2017 كنمط للممارسة. في سبتمبر/أيلول 2017، قدمت وزارة الصحة الصينية هذا النهج للرعاية المتكاملة المرتكزة على الأشخاص للدولة بأكملها. نحن نصف مزايا نموذج لوهو فيما يتعلق بمجالات العمل الأساسية واستراتيجيات التنفيذ المقترحة، كما أننا نلخص البيانات من تقييم للسنتين الأوليين من البرنامج. كذلك، فإننا نناقش التحديات التي تمت مواجهتها أثناء التنفيذ، والدروس المستفادة منها في أنظمة الرعاية الصحية الأخرى. نحن ننظر في كيفية تحسين التعاون بين المؤسسات، وكيفية تغيير سلوك السكان بخصوص استخدام الخدمات الصحية للمجتمع كنقطة اتصال أولى، وكيفية إدارة الموارد بفعالية لتجنب العجز في الميزانية. وأخيرًا، فإننا نوجز الخطوات التالية لنموذج لوهو وتطبيقه المحتمل، لتعزيز الرعاية الصحية في أنظمة الرعاية الصحية الحضرية الأخرى.

## 摘要

随着人口老龄化的加剧，大多数国家的慢性病病人对整合型卫生服务的需求也在不断增加。这种需求要求当前的卫生服务体系从根本上向着更加整合的服务提供模式转变。为帮助中国实现这一转变，世界卫生组织、世界银行和中国政府提出根据以人为本的一体化服务模式打造分级诊疗的卫生服务提供体系。深圳市罗湖区作为实践模板率先于2015年至2017年试点引入该模式。2017年9月，中国国家卫生和计划生育委员会向全国推介罗湖以人为本的整合型服务提供模式。我们描述罗湖模式的核心行动领域及相应的实施策略，并且总结该模式的第一个两年评估结果。我们讨论罗湖模式实施过程中所面临的挑战，以及它为其他卫生体系提供的经验。这些经验包括，如何促进机构间协作、如何改变居民的行为使其到社区卫生服务机构首诊、如何有效整合资源以避免医保基金赤字。最后，我们概述罗湖模式接下来的行动计划，以及该模式在加强其他城市卫生体系的潜在应用。

## Резюме

В большинстве стран по мере старения населения растет потребность в комплексном уходе для лиц, страдающих хроническими заболеваниями. Эта потребность требует изменения основополагающих принципов системы здравоохранения и сдвига в сторону моделей, в большей мере ориентированных на предоставление комплексных услуг. Для достижения этого сдвига в Китае Всемирная организация здравоохранения, Всемирный банк и Правительство Китая предложили внедрить многоуровневую систему предоставления услуг в соответствии с социально ориентированной моделью комплексного ухода. В качестве образца этот подход впервые был применен в районе Луоху города Шэньчжэнь в период с 2015 по 2017 год. В сентябре 2017 года Министерство здравоохранения Китая ввело принцип социально ориентированного комплексного ухода для всей страны. Авторы описывают характерные черты модели Луоху применительно к ключевым областям действия и предложенные стратегии внедрения этой модели, а также приводят итоговые данные, полученные в результате оценки первых двух лет проведения программы. Обсуждаются проблемы, с которыми медицинские работники столкнулись при ее внедрении, а также тот опыт, который может быть полезен для других систем здравоохранения. Авторы рассматривают такие вопросы, как улучшение взаимодействия между организациями, изменение поведения населения при использовании учреждений общественного здравоохранения в качестве пунктов первоочередного обращения за медицинской помощью, а также эффективное использование имеющихся ресурсов с целью исключения дефицита бюджета. Наконец, описываются дальнейшие этапы модели Луоху и ее потенциальное применение для укрепления здравоохранения в других аналогичных городских системах.

## Introduction

On 1 September 2017, China’s health ministry introduced a new approach to people-centred integrated care to the entire country.^1^ Called the Luohu model, the approach was pioneered in Luohu district of Shenzhen city. This development was a response to the problems faced by the existing health-care system in addressing the increased demands of delivering integrated care.^2^^,^^3^ Health-care systems worldwide are facing similar problems emerging from epidemiological transition and population ageing.^4^^–^^6^ Many people-centred integrated care programmes have been initiated, implemented and evaluated in high-income countries. While experience from other countries provides a useful basis for planning,^7^^,^^8^ the ability to achieve people-centred integrated care can be highly context-specific^8^^,^^9^ and there is a lack of knowledge about how to stimulate integrated care in low- and middle-income countries.^10^

The current system of health-care delivery in China is fragmented, hospital-centred and treatment-dominated, with little effective collaboration among institutions in different tiers of the system.^3^^,^^11^^,^^12^ In 2016, there were an estimated 231 million people aged 60 years or older in China, 16.7% of the population of 1 383 billion, and more than 100 million among them had at least one chronic noncommunicable disease.^13^^,^^14^ Predictions suggested that without health-care reform, China’s health-care costs in United States dollars (US$) would increase from 5.6% of gross domestic product in 2015 (US$ 592 billion of US$ 10 571 billion) to 9.1% in 2035 (US$ 2713 billion of US$ 29 810 billion).^15^ System reform was therefore viewed as necessary to avoid the risk of becoming a high-cost, low-value health-care system.

The World Health Organization (WHO) describes people-centred integrated care as health services that are managed and delivered so that patients receive a continuum of preventive and curative services according to their needs over time that is coordinated across different levels of the health-care system.^16^^–^^19^ Over the last decade, integrated care has been suggested as one strategy for promoting coordinated health-care delivery, improving quality of care and reducing costs.^20^^,^^21^ In 2016, the report *Deepening health reform in China* was published jointly by the WHO, the World Bank and the Chinese government.^15^ The report proposed strengthening health care in China through a tiered health-care delivery system in accordance with a people-centred integrated care model.

The introduction of the Luohu model set an example for urban areas in China to build people-centred integrated care delivery systems. This represented a big step in pursuing higher quality health care, better outcomes and more affordable costs for the population in China. In this paper, we describe the features of the Luohu model, discuss lessons learnt from its implementation and outline next steps for the Luohu model and its application in other Chinese urban health-care systems. We also provide suggestions on adapting the Luohu model in other low- and middle-income countries.

## The Luohu model

### Background

The Luohu model was a response to the needs of patients and their families in Luohu district (Health and Family Planning Commission of Shenzhen city, unpublished data, 2015). With a population of around 1.47 million in an area of 78 km^2^, Luohu is the most densely populated district of Shenzhen city, Guangdong province. In 2014, over 451 000 people were estimated to live with chronic diseases in Luohu (Gong F, Luohu hospital group, unpublished data, 2014). There was a city hospital with 2000 beds, five district-level public hospitals with a total of 1172 beds and 83 community health stations providing ambulatory care in the district. The growing size of the city hospital resulted in increasing numbers of patients attending. Since patients had greater trust in providers at the city-level hospital than the community health stations, they often sought services directly at the hospital despite receiving a lower reimbursement of medical expenses. Furthermore, many patients stayed in hospital for post-acute care rather than accessing this care in community health stations, because city- and district-level hospitals and community health stations operated independently and competed for patients. The government of Shenzhen city and Luohu district were concerned about the unmet needs of the population and the increased health expenditure associated with inappropriate hospital use and lengths of stay.

**Fig. 1:**
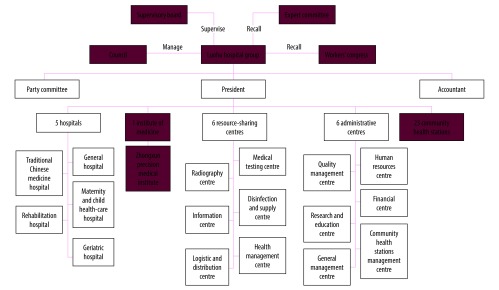
Organizational structure of the Luohu hospital group, Shenzhen city, China

In February 2015, the Luohu government initiated a health-care reform programme in cooperation with the local ministries in Shenzhen (the Health and Family Planning Commission, Ministry of Human Resources and Social Security, and Ministry of Finance). The stated goals of the Luohu people-centred integrated care model were better services, less illness, fewer hospital admissions and lower financial burdens. In August 2015, an integrated organization – the Luohu hospital group – was established, comprising five district-level hospitals, 23 community health stations and an institute of precision medicine. A council composed of government officials and representatives from local communities managed the group with the support of a local supervisory board, expert committee and workers’ congress. The group established six resource-sharing centres and six administrative centres (Fig. 1) by reorganizing the relevant centres of the previous 29 institutions, to improve the efficiency of both resource use and administration.

### Actions and strategies

The policy report *Deepening health reform in China* recommended eight core action areas: (i) primary health care to become the first point of contact; (ii) multidisciplinary teams; (iii) vertical integration; (iv) horizontal integration; (v) eHealth; (vi) integrated clinical pathways and dual referral systems; (vii) measurement and feedback; and (viii) certification and their accompanying strategies to achieve people-centred integrated care.^15^ The Luohu model implemented all the suggested core actions except certification (Table 1).

**Table 1:** Core actions and strategies to achieve people-centred integrated care in Luohu district, Shenzhen city, China

Core action area^a^	Implementation strategies^a^	Implemented?	Specific description in the Luohu model	Document reference	
Primary care as first contact	Patient registration	Yes	Residents in Luohu district are encouraged to sign a contract with a general practitioner voluntarily. The agreement defines a package of services, the service delivery process, and the rights and obligations of both patient and provider. Contract period is one year with a specific general practitioner. At the end of the period the patient can sign a contract with another general practitioner, which allows some element of patient choice.	Luohu government resolution no. 24 [2015]: Implementation plan for comprehensive reform of public hospitals in Luohu district	
	Risk stratification	Under preparation	Previous electronic information systems could not support risk stratification. Luohu hospital group is preparing to collect data for a risk stratification exercise based on disease burden, using a new computer application program.	NA	
	Gatekeeping	Yes	Patients are strongly encouraged to see their primary health-care provider before a visiting a hospital specialist. However, they are not formally required to do so. To promote patients’ use of family medical practices as the first contact, district-level hospitals assign specialists to work temporarily in community health stations.	Luohu government resolution no. 24 [2015]: Implementation plan for comprehensive reform of public hospitals in Luohu district	
	Ensuring accessibility	Yes	Home visits are provided for patients who sign a contract with a general practitioner, especially for the elderly people.	National Health and Family Planning Commission of Luohu district resolution no. 67 [2015]: Implementation plan for home visits in Luohu district	
Multidisciplinary teams	Team composition, roles and leadership	Yes	In community health stations, each primary care team consists of a general practitioner (leader), nurse, public health physician and health promotion practitioner and may also include specialist physicians (e.g. geriatrician, paediatrician, internist), pharmacist, nutritionist or psychologist. The roles of each member are clearly defined, with flexibility to adjust roles based on patients’ needs and the context.		Luohu government resolution no. 5 [2017]: Lessons learnt from the Luohu model to promote the construction of district hospital group in Shenzhen
	Individual care plans for patients	Under preparation	The hospital group is preparing to use care plans for high-risk patients identified by a risk stratification approach.		NA
Vertical integration	Definition of facility roles within a vertically integrated network	Yes	The Luohu model defines the roles of each component of the hospital group to ensure coordination. District-level hospitals are centres of excellence in technology and staff expertise, focusing on providing high complexity of care and valuable rescue care for life-threatening situations. District hospitals also provide technical assistance and training to community health stations. Community health stations focus on providing preventive care, rehabilitation, case management and medical care for common diseases		Luohu government resolution no. 24 [2015]: Implementation plan for comprehensive reform of public hospitals in Luohu district Luohu hospital group resolution no. 3 [2017]: Charter of the Luohu hospital group (revised version of 2017) Luohu government resolution no.5 [2017]: Lessons learnt from the Luohu model to promote the construction of district hospital group in Shenzhen
	Provider-to-provider relationships	Yes	In the hospital group, provider-to-provider relationships are strengthened through technical assistance and capacity-building. District-level hospitals are responsible to provide clinical technical assistance through training, education and joint consultations to physicians in community health stations. Meanwhile, physicians in community health stations are encouraged to get three months of training in the hospitals		
	Forming facility networks	Yes	The hospital group was established in the form of an independent corporation consisting of 23 community health stations, five district hospitals and an institute of precision medicine (which mainly provides diagnostic testing). A council of government officials and representatives from local communities was set up, to which the group are accountable to. Six administrative centres were re-organized using the resources of the respective centres in the former five district-level hospitals. Twelve centres provide resources and management for the whole group		
Horizontal integration	Integrating of different types of care	Yes	The multidisciplinary primary health-care teams include former health promotion staff from family planning stations, public health physicians from the Chinese Center for Disease Control and Prevention and specialists from hospitals. Teams work cooperatively with other members to provide preventive care, screening, diagnosis, treatment, rehabilitation and case management for patients. Six resource-sharing centres (human resources, quality management, financial, research and education, community health station management and general management; Fig. 1) allow for more efficient use of resources through reducing care overlap		National Health and Family Planning Commission of Luohu district resolution no. 4 [2016]: Implementation plan for appointing public health physicians to work in community health stations
E-Health	Integrated electronic medical records systems	Yes	The hospital group designed the Healthy Luohu computer application. By logging into their personal account, both providers and patients can access electronic health records systems		Luohu government resolution no. 24 [2015]: Implementation plan for comprehensive reform of public hospitals in Luohu district
	Communication and care management functions	Yes	The Healthy Luohu application allows patients to request an online appointment with a specific physician in all institutions. Staff in community health stations can make an online referral for patients to hospitals. The application is also easy for patients to check physician information and update registration and payment forms		
	Interoperability of e-health across facilities and services	Under preparation	Providers in hospitals and community health stations can view patient records in their own institution. Luohu hospital group is establishing regulations to allow the electronic systems to link across institutions securely and effectively		NA
Integrated clinical pathways and dual referral	Integrated clinical pathways for care integration and decision support	Under preparation	Clinical pathways are being created to standardize the treatment and referral pathways between providers		NA
	Dual referral pathways within integrated care networks	Yes	In the referral gateway model, patients referred from community health stations are expected to receive expedited care in the district-level hospitals. Down-referral, which allows referrals of patients from hospital to community health stations for rehabilitation care or follow-up, is incentivized by a new health insurance payment system in the Luohu hospital group		Luohu government resolution no. 24 [2015]: Implementation plan for comprehensive reform of public hospitals in Luohu district
Measurement and feedback	Standardized performance measurement indicators	Yes	The Luohu hospital group established a performance measurement system and makes annual self-evaluations. Indicators focus on measures of capacity-building of staff at community health stations (e.g. numbers of staff working in the community health stations, numbers of outpatients) and obtaining patients’ experiences		Luohu government resolution no. 24 [2015]: Implementation plan for comprehensive reform of public hospitals in Luohu district
	Continuous feedback loops to drive quality improvement	Yes	The results are communicated back to stakeholders at all levels, early positive results and challenges are identified. The hospital group is designing new strategies based on measurement results of the last two years		
Certification	Certification criteria for local and national use	No	NA		NA
	Targets for criteria and use to certify facilities	No	NA		NA

NA: not applicable.

^a^ Core action areas and implementation strategies suggested by the policy report *Deepening health reform in China*.^15^

First, under the Luohu model, patients are encouraged to sign a contract with a general practitioner based at a community health station and use him or her as the first point of contact with the Luohu hospital group. However, the gatekeeping system is not mandatory and allows an element of choice for patients.

Second, in community health stations each primary health-care team consists of essential members: a general practitioner, a nurse, a public health physician and a health promotion practitioner. Teams may also include a pharmacist, psychologist or other specialist physician (e.g. geriatrician, paediatrician, internist) according to the needs of local residents. General practitioners lead in developing team priorities, patient goals and care plans, and approve test orders, medication and referrals.

Third, the Luohu hospital group comprises 29 institutions at the community and district levels. In this vertical network, district-level hospitals focus on providing complex care and emergency care for life-threatening situations. Community health stations provide health promotion, preventive care, case management and medical care for common diseases.

Fourth, multidisciplinary primary health-care teams help to integrate different types of care. Health promotion staff was recruited from the former family planning stations to provide health education for patients. Public health physicians working at the Chinese Center for Disease Control and Prevention provide services such as, responding to and reporting infectious diseases and public health emergencies and monitoring domestic water supplies. 

Fifth, hospitals and community health stations previously used two different electronic information systems and providers could only view patient records in their own system. With the help of a Chinese internet company, Luohu hospital group designed a new computer application called Healthy Luohu, which all health-care providers can access. Patients too can access their own medical records online. 

Sixth, there is a referral gateway between community health stations and hospitals in the group. Patients can be referred from community health stations to hospitals for expedited care or can be referred back from hospitals for continuous rehabilitation care and follow-up within primary care. Patients referred via the gateway do not need to go through the hospital patient registration process and are given priority for care in the hospital compared with those directly accessing the hospital.

Seventh, the Luohu hospital group established a performance measurement system. The general management centre is responsible for making annual evaluations of performance using data collected by the information centre (Fig. 1). The results are communicated back to stakeholders to review their personal performance and identify problems which are then used to drive continuous improvement.

### Preliminary evaluation

**Fig. 2:**
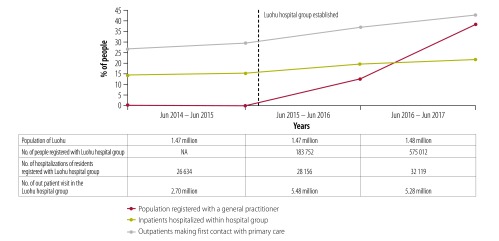
Use of integrated care in the Luohu hospital group, Shenzhen city, China, 2014–2017

**Fig. 3:**
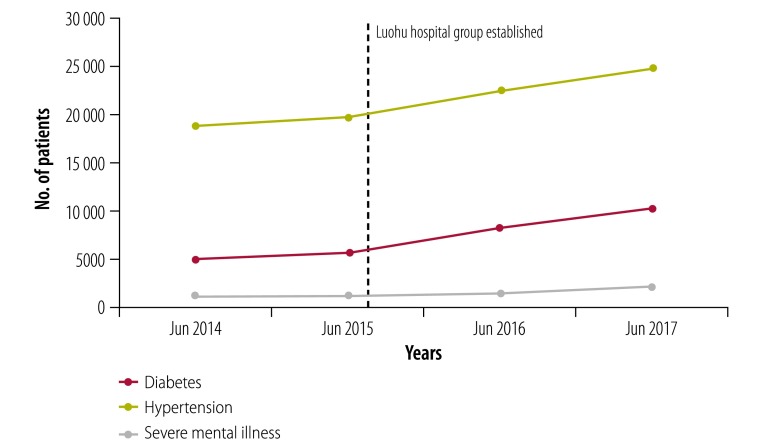
Number of patients under integrated case management by condition in the Luohu hospital group, Shenzhen city, China, 2014–2017

According to the annual self-evaluations of the Luohu hospital group, 575 012 residents (around 39% of the population) had signed contracts with primary health-care teams by July 2017. From June 2015 to June 2017 increasing proportions of the population used services in the Luohu hospital group rather than other hospitals outside the group after establishment of the integrated care programmes (Fig. 2). Increasing number of patients with diabetes, hypertension and severe mental illness are now under integrated case management (Fig. 3), which reflects greater collaboration between district-level hospitals and community health stations. From 2015 to 2017 the administration expenses of the whole group reduced by 19% (from US$ 30.0 million to US$ 24.3 million), and the average salary of staff in community health stations increased by 10% (from US$ 26 915 to US$ 29 607). Furthermore, a survey of about 80% of residents in 10 districts found that satisfaction with health care in Luohu district ranked first among all 10 districts in Shenzhen city.^22^

NA: not applicable.

Notes: Blue line is the proportion of the Luohu population who registered with a general practitioner within the Luohu hospital group. Light blue line is inpatient hospitalizations within the Luohu hospital group as a proportion of all hospitalizations of residents registered with the Luohu hospital group. Grey line is outpatient visits in community health stations as the first point of contact as a proportion of all outpatient visits in the Luohu hospital group.

Data source: Self-evaluations of the Luohu hospital group.

Data source: Self-evaluations of the Luohu hospital group.

The health ministry of China was satisfied with the results of the two-year preliminary evaluation in Luohu. Recognizing that it was a comprehensive model adopting and combining strategies from other initiatives, the ministry began introducing the Luohu model to urban areas nationwide on 1 September 2017.

## Lessons learnt

Despite promoting care integration within the hospital group and first point of contact at community health stations, the Luohu model provided several lessons to overcome challenges during implementation.

### Improving collaboration

The first challenge was how to improve collaboration between community health stations and district-level hospitals. Three reasons have been proposed for the fragmentation of services in China: (i) fee-for-service payments; (ii) fragmentation of financing; and (iii) more generous health insurance for inpatient than outpatient services.^23^ Other researchers suggested that effective care integration can be achieved without the need for the formal integration of organizations.^24^^–^^26^ However, the establishment of the Luohu hospital group created a strategy of integration across organizations and played a key role in removing these three barriers in the Luohu model. First, as illustrated by others,^23^ the health ministry of China has the responsibility for health care, but no means to control the provision of health services. The ministry cannot negotiate health insurance payment reform with the social security ministry for individual institutions, but the entire hospital group can. Luohu was the first place to implement a new health insurance payment policy in China called Global Budget, Balance Retained. The policy ended fee-for-service payment for providers, with incentives for increasing service volumes, rather than improving patient health outcomes.^27^ Instead, the challenge was to balance the incentives to the hospitals and community health stations to work co-operatively to strengthen preventive care and reduce demand for care. To avoid physicians acting to reduce services, the quality management centre of the group is responsible for supervising physicians’ practices. Second, the Luohu model integrates multiple sources of finances. Subsidies from the finance ministry for providing preventive care, health insurance funds from the social security ministry, out-of-pocket payments from patients and payments from other sources are all managed by the group’s financial centre (Fig. 1). Third, the Luohu model ended the higher reimbursement rate of inpatient services compared with outpatient services and incentivized patients to seek care first at community health stations. For example, in community health stations common drugs for chronic diseases are available at 70% of the prices in hospitals. Organizational integration and the innovative Global Budget, Balance Retained approach are exemplars for other urban health-care systems in China.

An important recommendation for adopting the model in other systems is that development and maintenance of a common frame of reference between organizations, professional groups and individuals, is essential to promote collaboration between different tiers of the health-care system.^26^ In Shenzhen, community health stations have been affiliated with district hospitals since 2011. This has provided a shared mission and management, and shared values that provide a foundation for mutual trust and collaboration across tiers. Trust needs to be built for collaboration between institutions in other health-care systems.

### Changing patient behaviour

The second challenge was how to change the behaviour of the population towards using community health stations as the first point of contact, rather than going to hospitals. In the Luohu model, four strategies were used to overcome this cultural challenge. The first strategy was capacity-building in community health stations. Technical assistance from district-level hospitals contributed to the improvement of care quality in community health stations. The second strategy was people-centred care in community health stations. For example, in response to the needs of elderly patients confined to bed, the community health stations provided home visits to avoid unnecessary hospital admissions and maintain patients at home, while reducing the burden of care on family members. The third strategy was ensuring adequate supplies of common drugs in community health stations. According to a study of 22 city-level hospitals in Beijing, one-third of patients attended hospitals solely to receive drugs (numbers not stated).^28^^,^^29^ In the Luohu model, district-level hospitals shared all drugs with community health stations, which reduced unnecessary outpatient visits to hospitals. Finally, health promotion staff in primary health-care teams have sought to improve health literacy in the population since establishment of the hospital group. The proportion of the population with basic health literacy in Luohu increased from 9.3% (136 710 of 1.47 million) to 21.3% (315 240 of 1.48 million) in the first two years of the programme.^30^ This compares with a national figure of 11.6% in a survey of 84 987 people in 2016.^31^ Health literacy enables people to increase control over their health and health determinants, while health promotion activities promote mutual trust between the population and staff of community health stations. We therefore believe that improving the population’s health literacy contributed to changing attitudes and behaviour about using community health stations in Luohu.

These four strategies could be applied directly to health-care systems in other urban areas of China. An increased supply of general practitioners in Luohu was also important for providing the capacity to support integrated care through the gatekeeper strategy. During the period 2015–2017, the number of general practitioners in the group increased from 89 to 194, based on offering higher salaries and training in task-shifting for some specialists. In 2017, there were 3.02 general practitioners per 10 000 residents in Luohu, compared with an average of 1.38 per 10 000 for the entire country.^22^ Policymakers in other health-care systems might consider general practitioner training of some specialists and task-shifting from general practitioners to experienced nurses and public health physicians to fill the general practitioner gaps in the short term.

### Reducing costs

The third challenge was how to avoid budget deficits in the first year. The goal of lower financial burdens has not been achieved in the first two years of the Luohu model. The 2016 global budget of the Luohu model was given by the total cost of health insurance for registered residents in the previous year, multiplied by the average growth rate of the health insurance fund in 2016. However, the average cost of integrated care per registered resident in the group increased from US$ 675.3 in 2015 to US$ 844.2 in 2016. The deficit arose because the global budget was based on medical costs in previous years, rather than the costs of all aspects of integrated care. Cost of preventive and other public health care, such as cancer screening programmes for residents older than 50 years and pneumonia vaccination for residents older than 60 years old, were not included. The finance ministry of Shenzhen city made up for the budget deficit of the hospital group by reorganizing health expenditure for public health providers.^32^ Before establishment of the hospital group, public health care was mainly provided by three kinds of facilities: specialized public health-care facilities (including disease prevention and control facilities, and health supervision facilities); primary health care facilities (community health stations); and hospitals.^33^^,^^34^ The ministry recalculated the budget of public health care in 2017 for the hospital group based on the care provided in 2016.

We suggest that finance ministries in other cities or regions rolling out such a model of care, need to consider public health-care expenditure when calculating global budgets for hospital groups, to avoid budget deficits in the first year.

## Next steps

There are two remaining steps in the application of the Luohu model. First, several strategies have not yet been implemented (Table 1), including risk stratification, individual care plans for patients, integrated clinical pathways for care integration, decision support and certification. The Luohu hospital group is preparing to implement a risk stratification exercise based on disease burden.^22^ Once high-risk patients have been identified, individual care plans will be made. Clinical pathways are being created to standardize the treatment and referral pathways between providers and to integrate care and support decision-making. Second, monitoring and evaluation is necessary to determine the effectiveness of the Luohu model over time. Despite the new self-evaluation system, more indicators related to people-centred care, population health and financial burden over the long-term are required. Although residents’ satisfaction with health care in Luohu district was high, their experience of integrated care was not a focus of the present study, even though it is an essential part of the Luohu model. Nevertheless, we are planning to use patient-reported experiences as a measure for integrated care to evaluate the Luohu model. Evaluation results, in turn, will influence the implementation of the remaining strategies or care integration.

Although the health ministry rolled out the Luohu model to other urban areas of China, it will take time before the model is implemented nationwide. From September to December 2017, more than 1500 policymakers from health and other social sectors in 321 cities received on-site training in Luohu. The concept and mechanism of the Luohu model were adopted by most cities in China. However, some strategies could not be implemented in some cities, due to lack of resources and lack of support from the of finance ministry and the social security ministry. For example, insufficient numbers of general practitioners may delay the development of primary health-care teams, while the health ministry cannot promote health insurance payment reform without coordination with the social security ministry. Some recent ministerial reforms in China provide government action to promote health-care system transition from disease treatment to integrated care.^3^ Instituted in 27 March 2018, such reforms are expected to improve health insurance payments and integrated care delivery in local health-care systems and promote application of the Luohu model.

Additionally, developing certification criteria and conducting certification nationally would assure external accountability for promoting implementation of the people-centred integrated care model.

## Conclusion

The preliminary evaluation of the first two years of the Luohu model supports the principle of capacity-building in community health stations and care integration in the district. The model has become national policy and is spreading rapidly. Application of the people-centred integrated care model in health-care systems in other parts of China will promote the transformation from a hospital-centred and treatment-focused health-care system to a people-centred and community-based integrated health-care system. Lessons learnt from the development and implementation of the Luohu model in China may have implications for other low- and middle-income countries that have health-care systems organized around hospital funding and activities and that lack well funded primary health care. Integrating the different levels of care into an overall system of people-centred care delivery provides an opportunity to improve the allocation of available health-care resources and manage the costs of delivering care in ways that are determined more by the needs of patients and less by a fragmented system structure.
